# A study of practical drawing skills and knowledge transferable skills of children based on STEAM education

**DOI:** 10.3389/fpsyg.2022.1001521

**Published:** 2022-10-13

**Authors:** Lan Yu, Yanfang Li

**Affiliations:** Collaborative Innovation Center of Assessment for Basic Education Quality, Beijing Normal University, Beijing, China

**Keywords:** children’s drawing, fine arts education, STEAM education, transferable skills, spatial perception, the sense of quantity

## Abstract

The STEAM education involves children’s ability to integrate and apply their knowledge of science, technology, engineering, arts, and mathematics. The application and transfer of interdisciplinary knowledge in practical activities is the structure of STEAM education. This study assesses children’s practical drawing skills and transferable skills based on the global features of their realistic figure drawing. The drawings incorporate the visual information and the multidisciplinary knowledge that children acquire. The assessment variables of the global features are observation perspectives, baseline, and comparison. The results showed that most children present their works through the front view. The children of different age groups show differences in express baseline and comparison features. Boys and girls show some variances in baseline features. Moreover, children are relatively unskilled at applying interdisciplinary knowledge in their drawings.

## Introduction

The education in the school fine arts curriculum is discipline-based art education (DBAE) (Broome et al., 2019). The DBAE educational model refers to children learning the foundational knowledge of fine arts, drawing practical skills, and appreciation ability of artworks through the fine arts curriculum (Chalmers, 2019). The purpose of discipline-based art education is to facilitate children’s skills in drawing creation. This education model responds to the problem of children’s techniques during drawing creation. STEAM education, by contrast, blurs the boundaries between different disciplines. The art curriculum in STEAM education develops children’s creative thinking, practical skills, and the ability to think independently (Perignat and Katz-Buonincontro, 2019). The art curriculum in this educational model is an activity-based program (Bazler et al., 2017). Teachers will provide children with thematic art creative activities in STEAM education. Children are encouraged to design the content of the artwork themselves, assemble the materials for the drawing, and search the methods for completing the drawings. The STEAM education aims to provide children with the practical skills and interdisciplinary knowledge to create artworks. Hence, children’s drawing education is training children’s drawing skills and fostering the application of multidisciplinary expertise and creative thinking training. This study evaluates children’s ability to apply interdisciplinary knowledge during drawing practice based on STEAM education.

The drawings present appearance information about the height, width, volume, texture, and color of objects (Ferretti, 2018; Winner, 2018; Foley and Bates, 2019). The appearance of objects present in children’s drawings is available by their visual estimation of the volume and quantity. Children can obtain the quantity sense from the mathematics curriculum (Booker et al., 2015; Albarracín and Gorgorió, 2019). Hence, the drawing creation process involves children acquiring both drawing skills and applying skills of interdisciplinary knowledge. The positional relationship, proportional relationship, and visual angle between objects are the factors that constitute the visual space features of a drawing (Briscoe, 2016; Ferretti and Marchi, 2021). The location distribution of objects in the drawing represents the position information, scale information, and shape information of the object that creators perceive in the life scenarios. Composition features mean the positional relationship between objects, showing the front of the direction, back, left and right, and up and down is compared to another. These compositional features build the sense of depth and field of vision in works. It is feasible to identify the creator’s observational habits and drawing capabilities from the position of the figures and objects in the drawings (Gao, 2018). The figures or objects close to the creators will appear in the most visible place in the drawings. The creators will also paint the clothing, decoration, volume, color, and other characteristic information of figures or objects in detail. The proportion of figures or objects far from the creator is smaller than those that are close. And these things will be placed at the back or far from the main elements of the drawing. Accordingly, this study assesses children’s drawing ability and application of interdisciplinary knowledge based on the visual space features of their drawings.

## Research background

The role of the fine arts in interdisciplinary STEAM education is to guide children in acquiring knowledge of science, technology, engineering, and mathematics in arts-themed activities. The arts-themed activities require children to sketch their pieces, learn engineering knowledge, prepare the materials for their creation, finish the artwork and present the achievements to classmates (Jeon et al., 2017; Herro et al., 2019; Barnes et al., 2020). These contents also illustrate that children have to acquire scientific, technological, engineering, and mathematical knowledge related to the subject of the creation before they complete the activities. The drawing creation process also forms part of the artistic activity. In the drawing creation process, children will learn to utilize the drawing techniques, design the appearance features of the figures, and apply interdisciplinary knowledge to form the spatial features in their drawings. It is possible to assess the capacity to apply knowledge across disciplines according to the observation perspectives, baseline, and comparison features from children’s drawings. Children have the awareness to apply interdisciplinary knowledge in arts-themed activities (Ngamkajornwiwat et al., 2017; Peppler and Wohlwend, 2018; Ahmad et al., 2021; Timotheou and Ioannou, 2021). However, the influences of interdisciplinary knowledge on children’s performance about the visual effects of drawings remain further assessed. For this reason, this study will assess children’s knowledge transferable skills based on the visual features of their drawings. The observation perspectives, baseline, and comparison features in children’s drawings are associated with their perception of spatial concepts (Vujakovic et al., 2018; Krichker, 2021). Understanding spatial concepts is also the foundation for children learning geometry (Stieff and Uttal, 2015; Young et al., 2018; Mix, 2019; Hawes and Ansari, 2020). The learning of geometry knowledge can assist children in establishing their cognitive abilities to classify, measure, and characterize figures (Research Group of National Mathematics Curriculum Standards for Compulsory Education, 2013). The representation of the distance and size features between different objects in a drawing needs to be based on children’s graphical cognitive ability to achieve. Children’s understanding of geometry also provides the basis for children’s representation of the three-dimensional features of objects in drawings. The spatial features display in children’s drawings represent their perception of spatial concepts. Therefore, this study assesses children’s ability to apply interdisciplinary knowledge based on the spatial characteristics of their drawings.

STEAM education aims to achieve children’s academic skills through practical activities (Quigley et al., 2017). The Chinese primary school mathematics curriculum standards also require teachers to design curriculum content based on real-life situations (Ministry of Education of the People’s Republic of China, 2012, 2022). For example, the mathematics textbook involves measuring the length of objects in real life and identifying the direction of buildings in real-life scenarios (Research Group of National Mathematics Curriculum Standards for Compulsory Education, 2013). The primary fine arts textbooks also include courses on observing natural surroundings. Children need to perceive changes in the color and appearance of plants under different weather conditions and sunlight (Research Group of National Fine Arts Curriculum Standards for Compulsory Education, 2012). These elements indicate that the current stage of compulsory education in China and STEAM education have the same educational purpose in teaching subject knowledge based on life practice. Paintings are the visual information that creators observe based on the objects’ location and shape features. The observation perspectives, compositional features, comparison features, and positional features in paintings are the components of the global features of the work (Kandel, 2016; Stanyer, 2020; Bunce et al., 2021; Goldstein and Cacciamani, 2021). When children create drawings, they need to use their visual senses to estimate the volume and location features of surroundings and then record these features in their drawings. The primary school mathematics curriculum in China involves training related to the visual estimation of the quality of objects and recognition of the orientation (Research Group of National Mathematics Curriculum Standards for Compulsory Education, 2013). This element also reflects that Chinese children can estimate the appearance of objects. However, children’s ability to translate appearance features into drawings need to further assessment. This study therefore assesses children’s ability to apply interdisciplinary knowledge based on the viewing perspective, baseline features and comparison features of their drawings.

## Materials and methods

### Content for the research

This study assesses children’s knowledge transferable skills through the observation perspectives, baseline feature and comparison feature of their drawings. The experimental component of the study consisted of a drawing creation task. The drawing task requires asking children to draw a piece of work contain persons and a real-life scene. There is no limitation on the gender and age of the people in the drawing. Before creating drawings, children need to learn the characteristics of the structure, color, volume, and texture of objects associated with life scene theme. Objects from life scenes are familiar and accessible to children. Children can draw regarding the actual appearance of objects. These observations and drawing processes also ensure that the children’s work is closer to the requirements of the test task. Realistic drawing is a record of people’s visual experience. In drawing creation, children need to show the observed features of shape, height, texture, texture, and location relationship of objects in their works. Therefore, children’s knowledge transferable skills in this study is assessed by the composition, structure, and location relationship features between objects in their drawings.

### Participants

The total number of children who participated in the test was 1,000, including 526 girls and 474 boys. These children’s intelligence levels are within the normal range. The cultural environment, educational level, and economic development level affect the academic skills of children (Rosselli and Ardila, 2003; Ozer, 2009; Becker et al., 2013). To avoid the effects of these factors on this test we choose children from the same city as the study participants. The content of the textbooks and the curriculum used in schools was also consistent among the children who participated in the test (Ministry of Education of the People’s Republic of China in 2010, 2019 and The Education Department of Jilin Province, 2018). All the children who participated in the test were between the ages of 7 and 12, with 282 children aged 7–8 years, 398 children aged 9–10 years, and 320 children aged 11–12 years (Table 1). The classification of age groups is based on children’s academic ability and cognitive level. These children were classified into three age groups based on the content of their school mathematics curriculum and the content of the Chinese primary school mathematics curriculum standards (Ministry of Education of the People’s Republic of China, 2012 and Research Group of National Mathematics Curriculum Standards for Compulsory Education, 2013). Furthermore, the drawing data used for this study were completed by children in six-year public elementary schools in China. All of these students were enrolled in schools that had fine arts classes. Therefore, the children who participated in the test had experience in drawing before the test.

**Table 1:** Gender and age distribution.

	Gender		Age			Total
	Girls	Boys	7–8 years old	9–10 years old	11–12 years old	
N	526	474	282	398	320	1,000

### Data preparation

Before the drawing test, children and their teachers have been told the content and purpose of the drawing test. Children were required to complete the drawing test independently within 40 min. They were not given any hints about drawing techniques, material application, or creative ideas during the test. This step ensures that their drawings more accurately reflect their drawing abilities.

### Materials

Children utilize the A4 (210 mm × 297 mm) paper during the drawing task. Using A4 size paper can help children to better complete the drawing. The smaller paper, such as A5 and B6, has a limited drawing area. This condition will disturb children from drawing the details of figures or scenes. Oversized drawing paper (e.g., B4, A3 paper) has too much blank space. This condition may cause children to be unable to complete drawing within the time specified. Children’s drawings may show incomplete scene content. As for drawing tools, children can choose pens according to their preference during the test. Different types of paintbrushes hold correspond to lines and color features. For instance, watercolor pens present thicker lines. Pencils, colored pencils, pens, and ballpoint pens deliver fine lines. On the one hand, children may choose brightly colored watercolor pens to highlight the main content of the drawing. For example, emphasize the contour lines of the figures and objects in the drawing. On the other hand, some children may prefer to use pencils, colored pencils, pens, and ballpoint pens to show the detailed features of the objects in the drawing. Children may use these drawing tools to express the details of the plant’s leaf veins, flower stamens, and the figure’s eyebrows and hair. Watercolor pens present a higher brightness of color than colored pencils, pencils, pens, and ballpoint pens. Some children may tend to create brightly colored or light-toned works. This study assesses the shape and position of objects according to children’s viewing perspective. The color and texture of the drawing materials do not determine the positional and shape characteristics of the objects in drawings. Also, the ability to apply material is not the evaluation indicator for this study. Therefore the color effects presented by different painting materials will not affect the results of this study. If the children’s drawing tools are specified in advance, it may change their original creative drawing habits. Limiting drawing tools may affect the expression of textural features of lines and detailed features in children’s work. Drawing tools familiar to children will reduce the time it takes for children to adjust new drawing materials. Allowing children to choose drawing materials according to their habits may lead them to participate more rapidly in creating paintings.

### Coding

Drawings are result from the integration of information about the appearance features of children’s observation, such as the observer’s angle of viewing the object and the position relationship between the observer and the observed object. The data evaluation in this study relied on assessing the observation perspective, the position of the baseline, and the comparison relationship between the objects and the figures in children’s drawings (Table 2).

**Table 2:** Coding of global features.

Global features		Examples
Observation perspectives	Front view 1	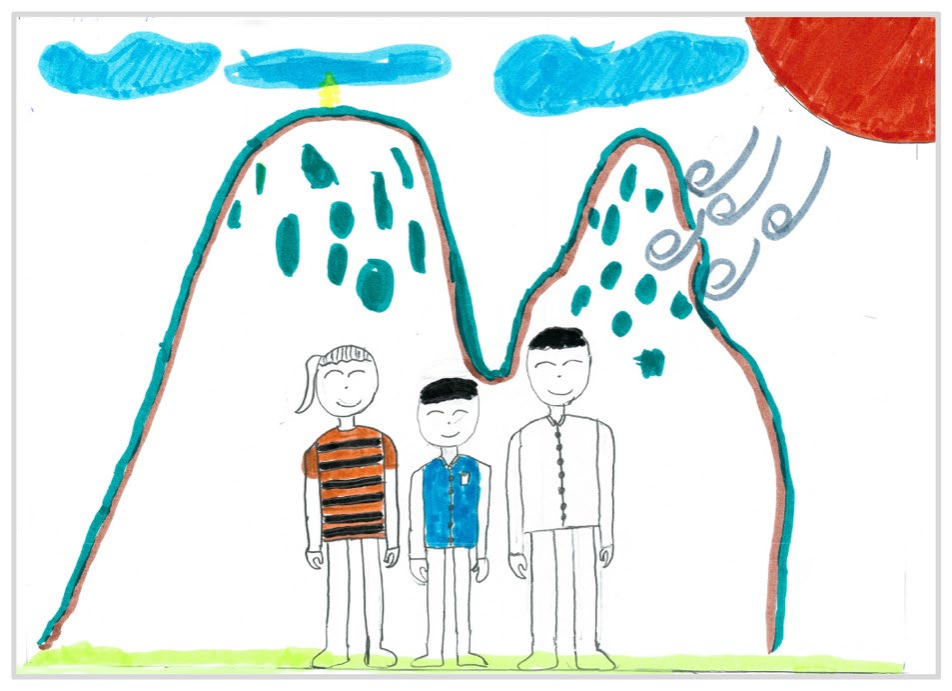 Front View 1
	Side view 2	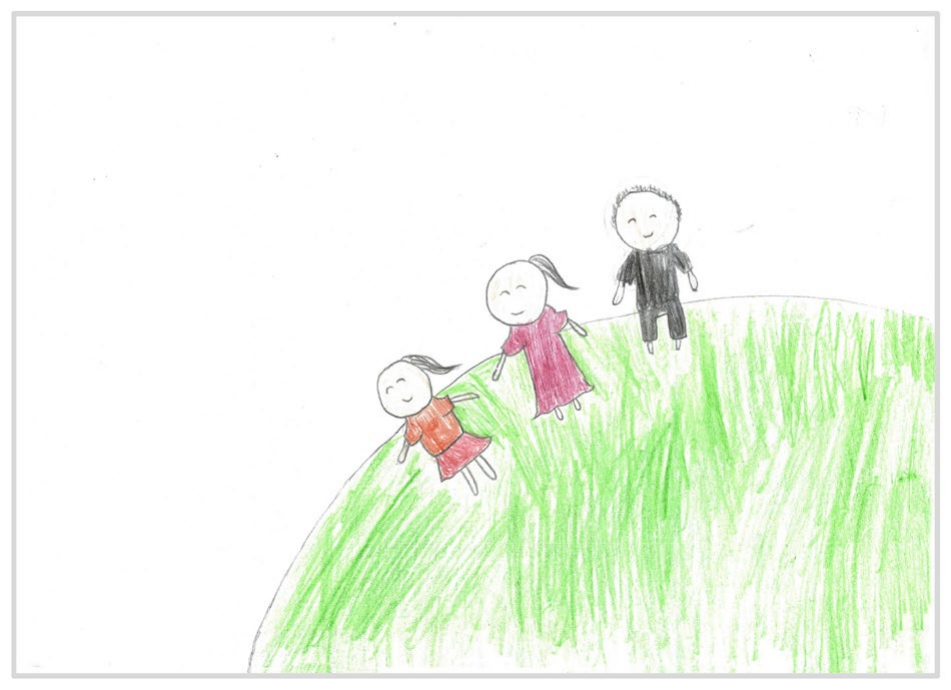 Side View 2
	Plan view 3	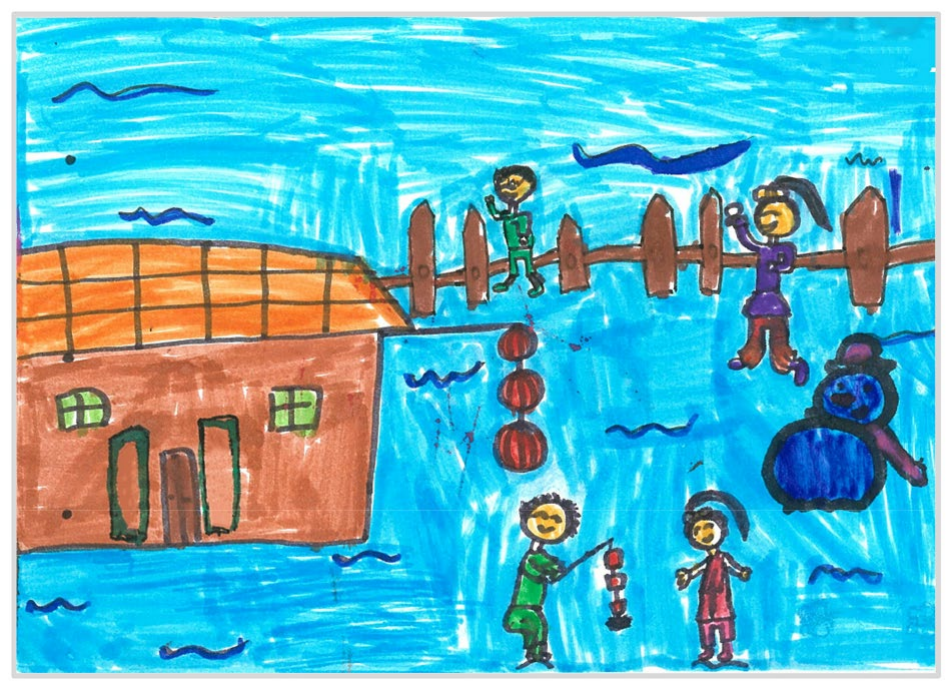 Plan View 3
	Upward view 4	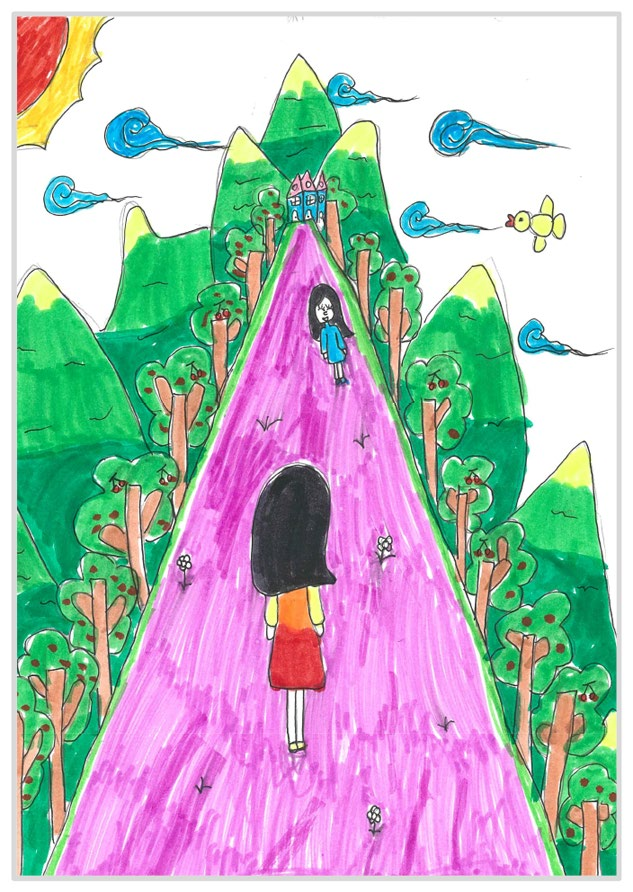 Upward View 4
	Mixed view 5	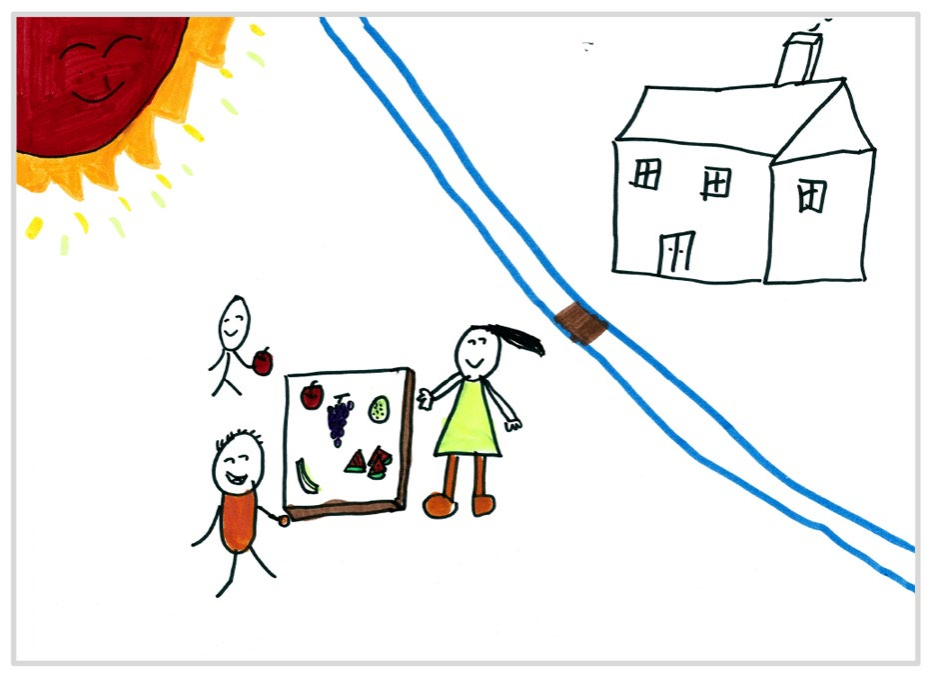 Mixed View 5
Baseline	Without baseline 1	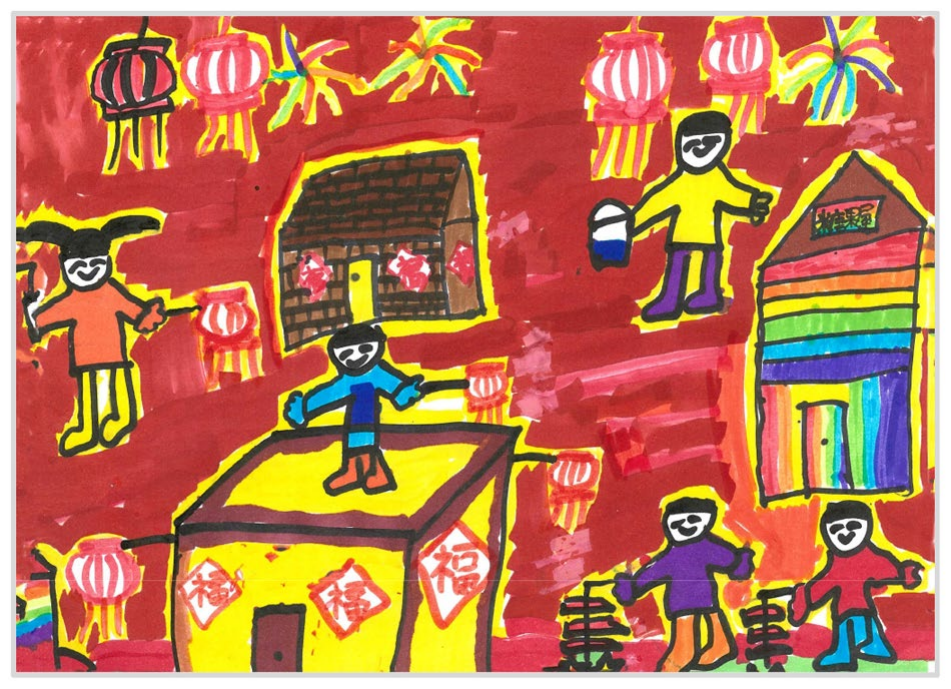 Baseline 1
	Objects drawn on baseline 2	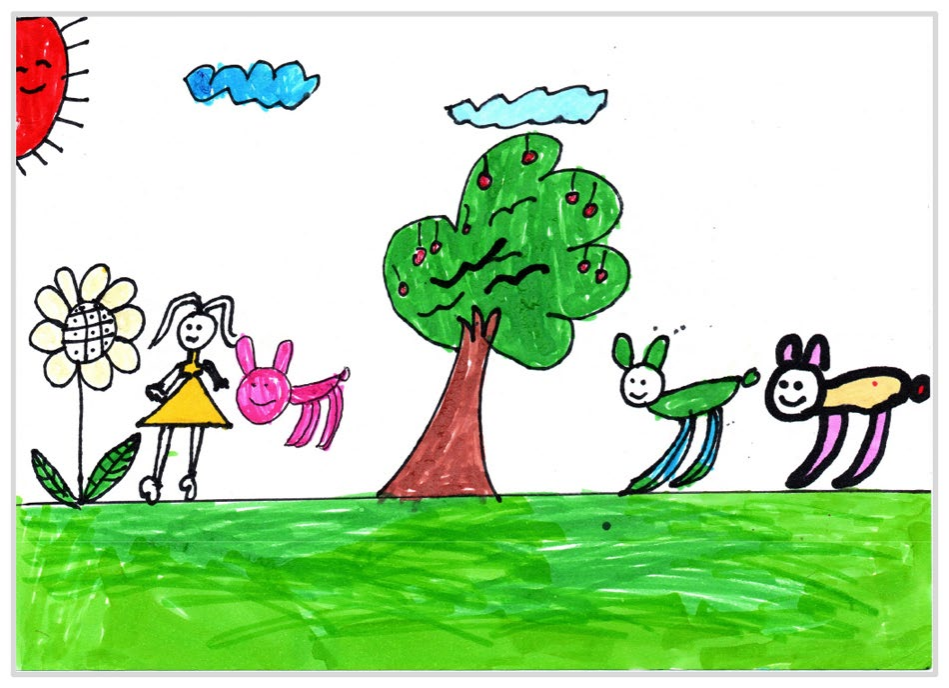 Baseline 2
	Baseline divided the space area 3	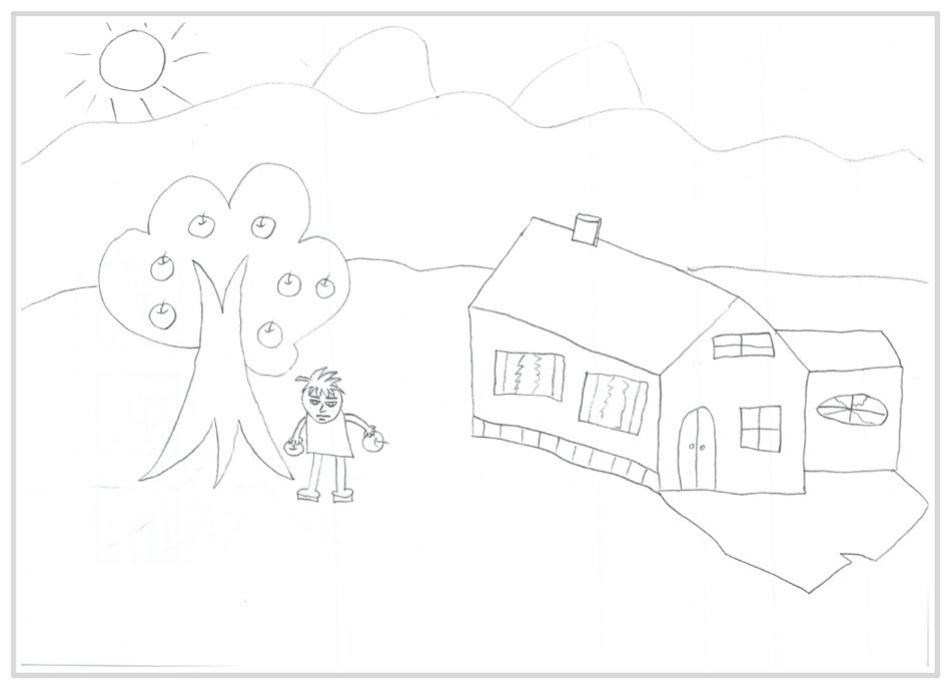 Baseline 3
Comparison	Wrong comparison 0	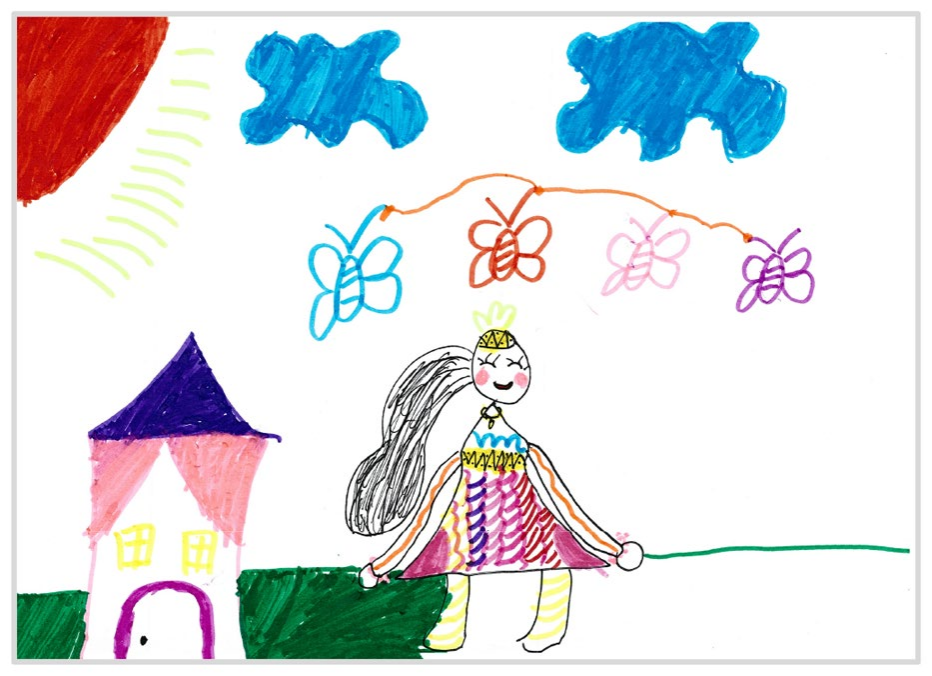 Wrong Comparison 0
	Distance comparison 1	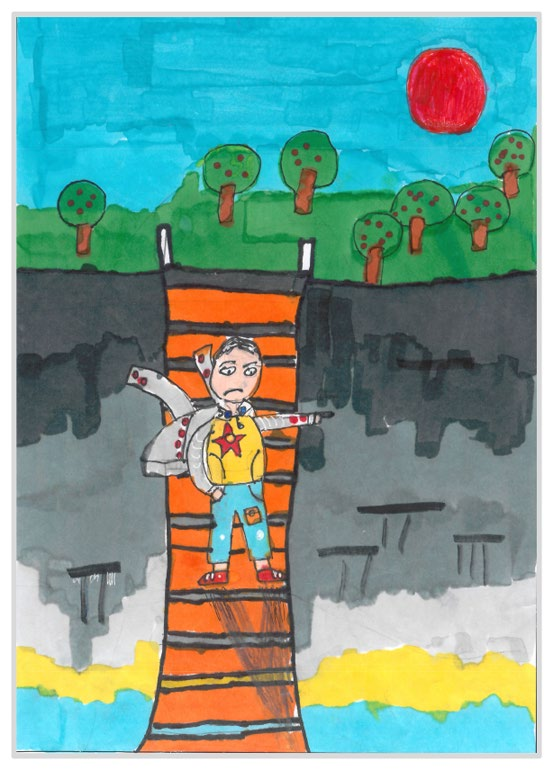 Distance Comparison 1
	Proportion comparison 2	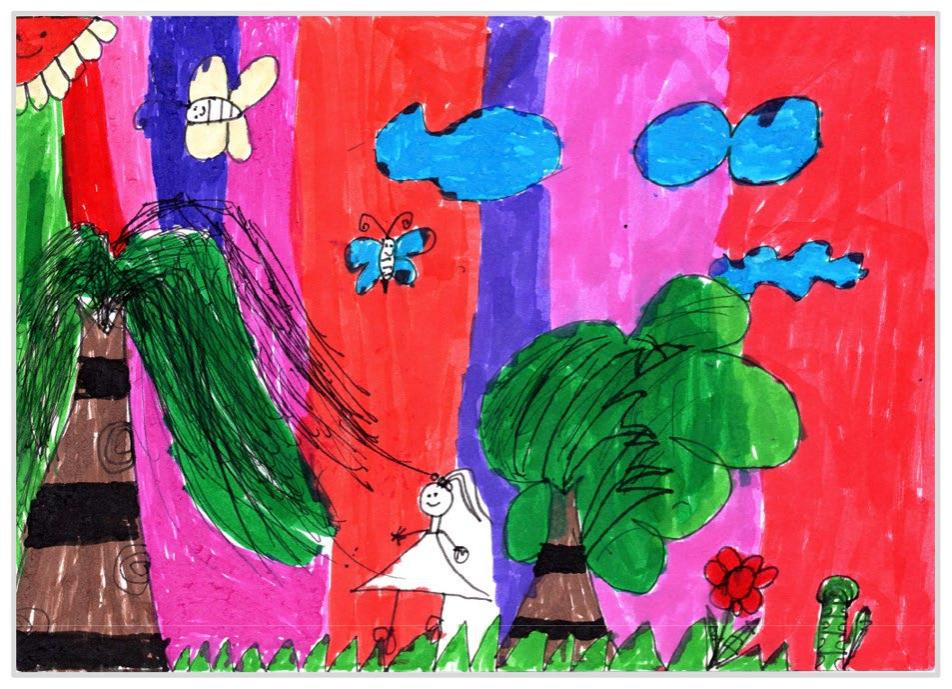 Proportion Comparison 2
	With distance and proportion comparison 3	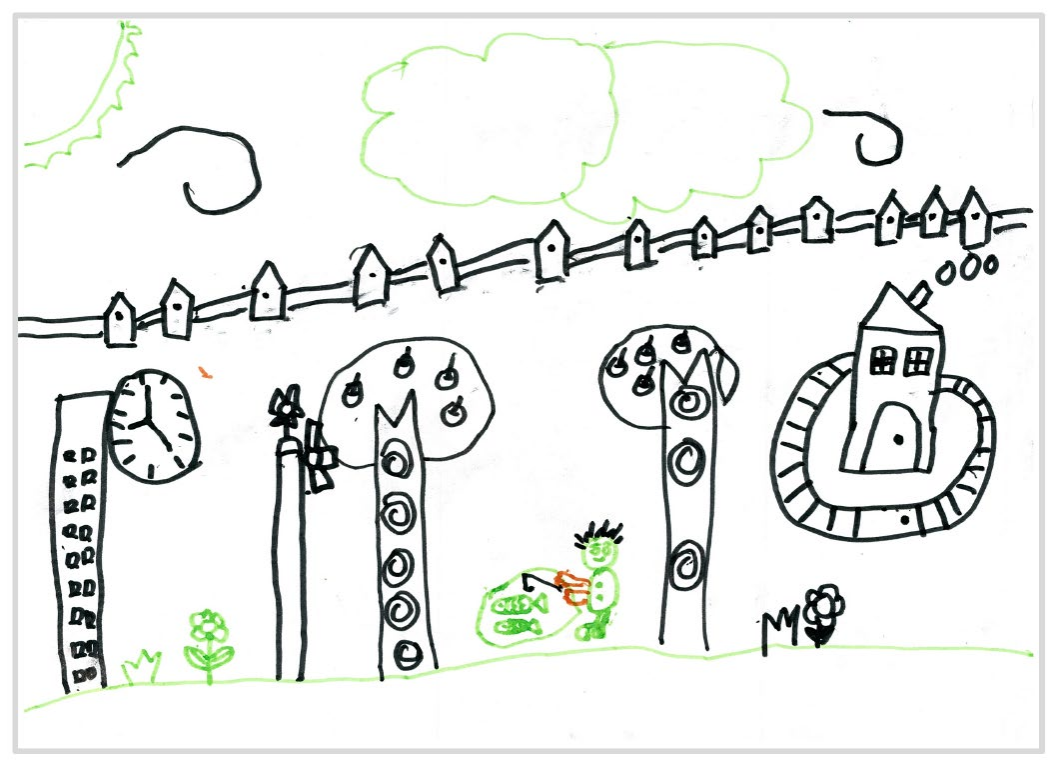 Distance and Proportion Comparison 3

The types of observation are classified according to the person’s observation perspective of objects in space (Talipov and Aliev, 2021 and Talipov, 2021). The observation perspective includes front view, side view, plan view, upward view, and mixed view. The child obtains the front view from the frontal view of the objects or figures. For example, the figures and the mountain in “Front View 1” are created based on the front view (Table 2). The position and orientation information of the figures in “Side View 2” indicates that the creator observes the characters and environmental scenes from the right side (Table 2). Moreover, the figures and the lawn are mainly located on the right side of the work. Therefore, “Side View 2” is classified in the category of the side view. The plan view is obtained by looking from the top to the bottom of the object. Drawings with plan views features will have baselines or objects that divide the drawing area. Also, the ground plane area in these works is larger than the facade area. For example, “Plan View 3” in Table 2, the fence divides the space between the ground plane and the facade. The ground plane area in “Plan View 3” is larger than the elevation area. The observer obtains an upward view from the bottom to the top of the object. The visual effect of the objects close to the creators is more prominent than those far away from them. Also, vanishing points may appear in such drawings. The vanishing point is formed by two or more lines representing parallel lines stretching toward the horizon line until they converge. “Upper View 4” in Table 2 shows a drawing created from an upward view. The two figures in the work jointly present foreshortening effects. The edge lines on either side of the road stretch toward the horizon line until they converge to form the vanishing point of the image. In addition, for children who cannot represent or distinguish viewing perspectives, at least two kinds of viewing perspectives may be present in their works. This type of works is classified as mixed view drawings. For example, “Mixed View 5” in Table 2 shows a drawing with mix view. Children create the figures in this work base on frontal observation. However, the viewpoint of the objects close to the figures is constructed based on the plan view. There are two perspective views present in this drawing. Thus, this drawing is categorized as mix view.

The role of baselines in children’s drawings is to create and divide different painting spaces (Morrison, 2013; Strauss, 2021). Baselines are also representative features of children’s cognition of painting spatial. Some children have a weak ability to express the ground features in their works (Burkitt et al., 2003; Terton et al., 2022). This issue will cause the missing baseline in their drawings. Other children capable of expressing ground features may draw objects on the baseline or remove objects on either side of the baseline (Burkitt et al., 2019). The baselines are the children’s understanding of the concept of space. Baselines express the children’s ability to represent three-dimensional space within a two-dimensional space. The baseline location is evaluated in three aspects: without a baseline, objects drawn on the baseline, baseline divides the space area. The lack of a baseline in pieces indicates that the child did not create the drawing with a sense of dividing the ground plane and facade area. “Baseline 1” in Table 2 is a drawing without a baseline. The background of this drawing is red. There are no lines that distinguish the ground plane from the facade area. Thus, this drawing represents a two-dimensional space feature. The lack of three-dimensional space also contributes to the lack of visual depth in the drawing. Depth is related to the front-to-back position of different objects in pieces. So the lack of baseline in the drawing also causes the positional relationship between objects to blurring. Some works contain the lines used to divide the drawing area. However, figures or objects in these drawings are arranged on a baseline. This type of work shows that children have a sense of representing the ground plane. Their awareness of the facade space is limited. “Baseline 2” in Table 2 shows an example of figures and objects arranged on a baseline. The child who created this drawing uses the baseline as a tool to support objects and figures that can be stable on the ground. However, this drawing lacks a description of the object’s front and back position relationship. So this drawing only represents two-dimensional visual space. A baseline that divides the space area means a line dividing the drawing’s space area. The figures and objects in pieces are located above and below the baseline. These drawings display the existence of both flat and three-dimensional space. It also shows that the children who created this type of drawing are aware of creating three-dimensional space in drawings. For example, a line behind the house and tree divides the ground plane from the facade drawing area in “Baseline 3” (Table 2). This line separates the house, tree, or figure from the mountain and sun in the drawing. Therefore, “Baseline 3” is classified as a drawing with a baseline that divides the space area.

The position and proportional relationship between different objects constitute the visual–spatial effect of the drawing. The position and proportion of different objects are related to the spatial depth of the drawing (Farmer et al. 2018; Terton et al., 2022). For example, objects in works close to the observer have a larger volume than those far away. The position and proportional relationship between different objects is also a sign to distinguish the close and distant view of the drawing (Metin and Aral, 2020). The comparison assessment is divided into four types: the wrong comparison, distance comparison, proportion comparison, and distance and proportion comparison. The comparison in this study assesses the relationship between the key elements such as plants, animals, people, houses, and mountains. However, this study will not assess the sky elements such as sun, clouds, stars, moon, and rainbow. The objects on the same horizontal line have opposed to realistic proportion features that will be classified in the category of the wrong comparison. For example, the figure and the house in “Wrong Comparison 0” are on the same horizontal line and have the same height (Table 2). There is a wrong proportional relationship between the house and the figure. Therefore, this work is classified in the category of the wrong comparison. The distance comparison is related to the contrast between objects’ front and back positions. The drawing only contains the relationship between the front and back positions. If there is a distance comparison between objects in the drawing and there is no proportion comparison between different types of objects on the same horizontal line, then the drawing is classified as a distance comparison item. “Distance Comparison 1” in Table 2, there is a distance relationship between the figure and the trees. So this drawing is classified as a distance comparison. If drawings only contain a proportional comparison feature between different objects on the same horizontal line, this type of drawing will be classified in the category of proportional comparison. “Proportion Comparison 2” in Table 2 shows a piece of drawing example of a proportional comparison between plants and figures. The drawing includes both distance and proportional features belonging to the distance and proportion comparison category. “Distance and Proportion Comparison 3” in Table 2, there is a distance comparison between the fence and the plants, figures, and houses. There is a proportional comparison between the figure, the house, and the plant. So this drawing belongs to the category of distance and proportion comparison.

## Data analysis

Most of the children represent the figures, objects, and scene environment of the drawing based on the front view (Table 3). Only 0.3% of the children created their works by drawing the sides of objects or people. The observation view results show that plan views, and upward views accounted for 3.3 and 4.4% of the total number of drawings. This result indicates that most children have more experience observing the frontal view of objects than other observation perspectives. Besides, the statistical results show that 11.8% of the children’s drawings have mixed views. These results indicate that children have less express experience with the sides of objects or figures in their works.

**Table 3:** Distribution of global features.

Features	Types of features	Percent %	Frequency	Total
View	Front view	80.2	802	1,000
	Side view	0.3	3	
	Plan view	3.3	33	
	Upward view	4.4	44	
	Mixed view	11.8	118	
Baseline	Without baseline	39.8	398	
	Objects placed on the baseline	28.1	281	
	Baseline divided the drawing areas	32.1	321	
Comparison	Without or wrong comparison	35.8	358	
	Proportion comparison	34.4	344	
	Distance comparison	10.9	109	
	Distance and proportion comparison	18.9	189	

The role of the baseline in children’s drawings is to divide the work into different visual areas. Children use baselines to divide the area of ground and sky. The ground element in the drawing is the supporting area to make the objects placed in a stable condition. The ground element is also the ground plane of work. The sky element represents the facade of the work. The ground plane and the facade compose the three-dimensional visual space of the work. The comparison features of objects in works that accurately represent the baseline are more evident than in works of others baseline types (Table 4.). Although more works without a baseline type show the distance comparison features than in the other two baseline types of works, children who did not draw a baseline were weak in expressing both the distance and proportion features of objects in their works. This result shows that children who did not draw a baseline have limited cognition in expressing the comparison features in work. The distance comparison feature determines the spatial depth of the work. The proportion relationship determines the shape features of objects. Most of the children who did not draw a baseline only show one comparison feature of the proportion and distance comparison features (Table 4.). This result shows that the ability of these children to organize the visual–spatial relationship of the complete entire drawing still needs to improve. Drawings without a baseline mean that the objects and figures in work lack the ground that supports them to keep stable visual conditions. The visual effect of these drawings may show all objects being suspended in the air. Combining the results of Table 3 and the rules of painting creation practice shows that works with baseline have more obvious three-dimensional spatial effects than works without baseline. Besides, the statistical results of all children’s works show that most works do not display baseline features (Table 4.). Thus, it is difficult for most children to represent three-dimensional space in their drawings.

**Table 4:** Percentages between different baseline types and comparison types.

Items	Without baseline	Objects placed on the baseline	Baseline divided the drawing areas
Without or wrong comparison	11.8	18.5	8.1
Proportion comparison	37.2	49.8	17.4
Distance comparison	40.7	23.8	35.2
Distance and proportion comparison	10.3	7.8	39.3

Age, gender, and baseline features were categorical data (Ahi, 2017). For this reason, age-related and gender-related differences in children’s performance of baseline features were calculated using the Pearson Chi-squared test. Children of different genders and age showed significant differences in the baseline features of their works. The results (*χ*^2^ = 7.236, *p* = 0.027 < 0.05) showed a significant difference in the baseline performance of different genders of children in the works (Table 5.). Comparing the percentage results of boys and girls showing baseline features in the drawings showed that more girls than boys did not draw baselines. Also, more boys than girls drew the other two types of baseline features. Thus, the above results show that boys are better than girls at expressing baseline features. At the same time, the three-dimensional spatial features of boys’ drawings are more evident than those of girls. Objects placed on the baseline mean that the child can divide the drawing area of the work, but the location description of the baseline is not accurate. The baseline divided by the drawing areas represents the child can separate the drawing area and draw the correct position of the baseline. Comparing these two types of baseline statistic results shows that more children accurately drew the baseline location than those who did not accurately draw the baseline. This result indicates that most of the children can accurately delineate the regional features of the ground and sky by using a baseline.

**Table 5:** Variance analysis between gender, age group and baseline.

Items		Baseline %			Total %	*χ* ^2^	*p*-value
		Without baseline	Objects placed on the baseline	Baseline divided the drawing areas			
Gender %	Boys	35.65	28.90	35.44	47.4	7.236	0.027^*^
	Girls	43.54	27.38	29.09	52.6		
Total %		39.80	28.10	32.10	100		
Age %	7–8 years old	37.59	39.36	23.05	28.2	35.283	0.000^**^
	9–10 years old	36.43	24.87	38.69	39.8		
	11–12 years old	45.94	22.19	28.10	32		
Total %		39.80	28.10	32.10	100		

* *p* < 0.05;

** *p* < 0.01.

The results (*χ*^2^ = 35.283，*p* = 0.000 < 0.01) showed that children of different age have differences in performing baseline features (Table 5). Comparing the percentages of the three age groups showed that children between ages 9 and 10 years performed better at baseline than the other two groups (Table 5). Most children between ages 7 and 8 years draw objects on the baseline. Only a few children can express baseline accurately. These two results indicate that children aged 7–8 years can use the baseline to divide the space between the ground and the sky of the work. However, their ability to express baseline features still requires further training. Most children aged 9–10 years can accurately draw baselines. Children aged 9–10 years drew objects on the baseline were the fewest. Therefore, children between ages 9 and 10 years can accurately represent baseline features. The number of drawings without a baseline was higher in the pseudorealism stage than in the other two drawing stages. This result indicates that children in this stage need to train their ability to express baseline features.

**Figure 1:**
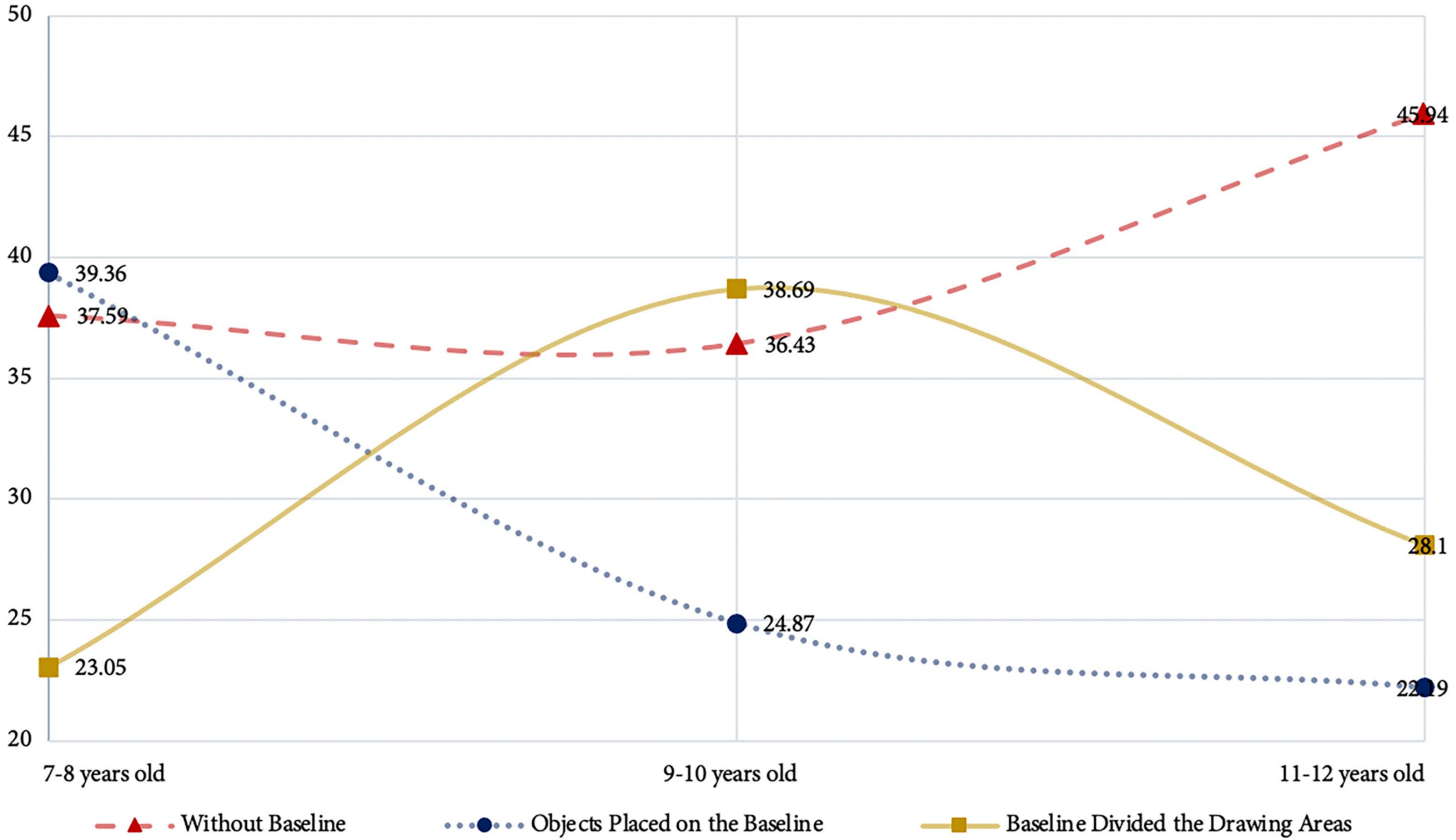
Trend chart in baseline features at different age stages.

The number of children aged 9–10 years old who did not express baseline features was less than the number of children aged 7–8 years old. This result suggests that children aged 9–10 years old have better at performing baseline than children aged 7–8 years old. There is a clear increasing trend in the number of children who do not draw a baseline during children between ages 11 and 12 years (Figure 1). This part of the data indicates that children between ages 11 and 12 years have a lower ability to draw baseline than those children aged 9–10 years old. Moreover, the number of children aged 11–12 who did not display baseline in drawings is more than the number of children aged 7–8 years old. Thus, the baseline performance ability of some children after 10 years old will show a downward trend with the increase of children’s age.

Age-related differences in children’s performance of comparison features were calculated using the Pearson Chi-squared test. Children of different age showed significant differences (*χ*^2^ = 36.991，*p =* 0.000 < 0.01) in the performance of comparison relationship between objects in drawings (Table 6). Most children have a weak ability to express the comparison features in the works. 34.4% of the children showed the proportional comparison features in drawings (Table 6). For children who can paint the comparison features, their ability to draw proportional comparison features is higher than that of distance comparison features. Also, only 18.9% of the children can express both distance and proportion features in their works (Table 6). The proportional relationships in the drawing are related to the width and height of the object. Length and width compose the effect of the two-dimensional space of the drawing. The distance relationship between objects constitutes the effect of the three-dimensional space of the work. The distance relationship between objects also represents the depth of field of the drawing. Therefore, the results of the comparison features indicate that children of all ages have a weaker ability to express the distance comparison feature in the drawing. This result also shows that children’s ability to represent works in three-dimensional space is weak.

**Table 6:** Variance analysis between age stages and comparison.

Features	Types of comparison	Age %			Total %	*χ* ^2^	*p*-value
		7–8 years old	9–10 years old	11–12 years old			
Comparison %	Without or Wrong Comparison	48.23	30.40	31.56	35.80	36.991	0.000^**^
	Proportion Comparison	33.33	33.42	36.56	34.40		
	Distance Comparison	6.38	14.32	10.63	10.90		
	Distance and Proportion Comparison	12.06	21.86	21.25	18.90		
Total		28.2	39.8	32	100		

** *p* < 0.01.

48.23% of the children between ages 7 and 8 years do not present or present the wrong comparison features (Table 6.). In the works showing the comparison features, 33.33% of the children between ages 7 and 8 years can indicate the proportional comparison relationship between objects (Table 6). Only 6.38% of children can show the distance comparison features between objects (Table 6). Thus, it shows that most children between ages 7 and 8 years have difficulty expressing the size and distance relationship of objects. Children aged 7–8 years old who can represent the comparison feature of objects have difficulty representing the distance comparison features of objects. Children aged 9–10 years old with the highest performance accuracy compared to the three age groups. Also, the number of children who displayed both distance and proportion features at aged 9–10 years old was higher than in the other age group. Therefore, children between ages 9 and 10 years with better performance among the three age groups.

**Figure 2:**
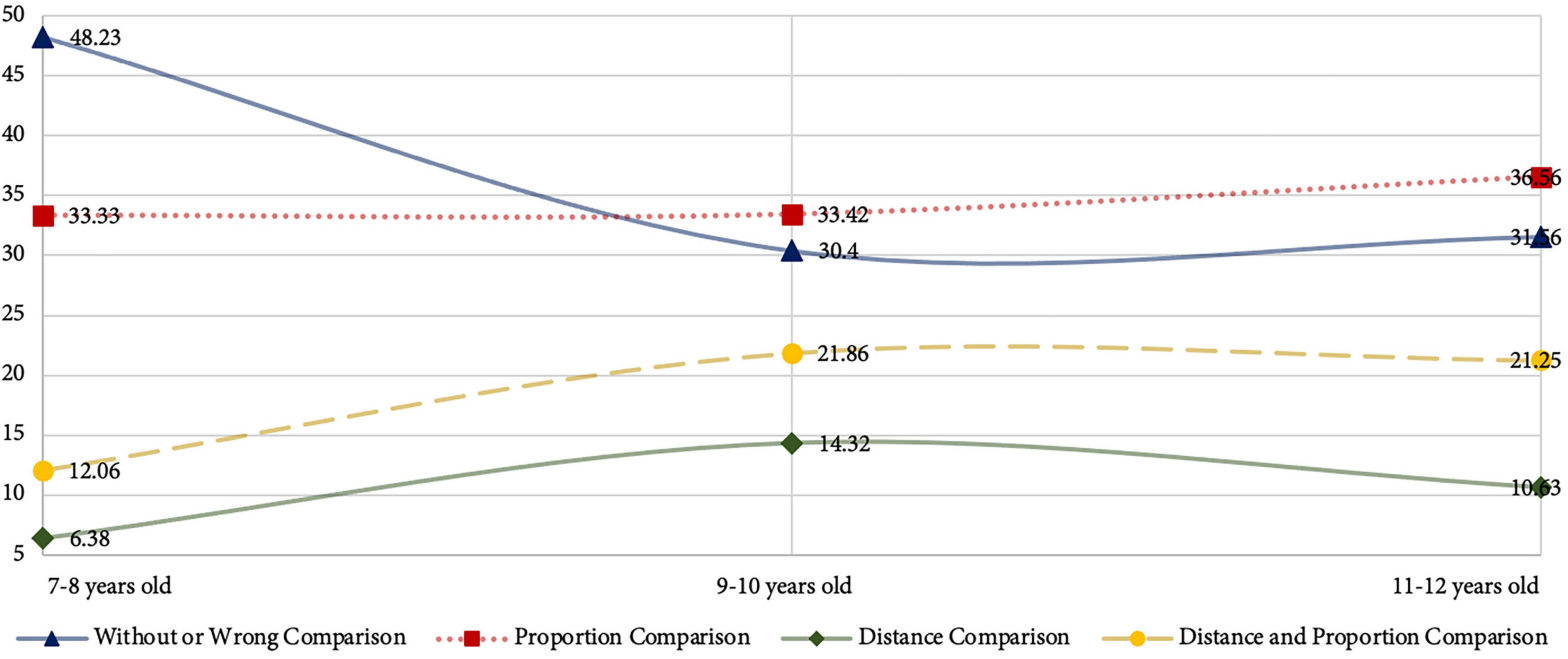
Trend chart in comparison features at different age stages.

The results of comparison features of the drawings in the three age stages show that children’s ability to express distant comparison increases with age from 7 to 8 years old stage to the 9–10 years old stage (Figure 2). There is a decreasing trend in children’s ability to express distant comparison features from the 9–10 years old stage to the 11–12 years old stage (Figure 2). The ability to draw proportional comparisons improves with the age of the children. Among the works that display comparison features, the most frequent feature shown by children is the proportional feature. The distance and proportional relationship between objects represent the spatial visual effect of the work. The distance comparison and volume of the objects compose the depth of the drawing. The proportional feature constitutes the relationship between the size of the objects and the plane effect of the drawing works.

The combination of these aspects shows that children capable of expressing comparison features can better draw the size relationship of objects and the overall plane effect of the drawing works. However, they have a weak ability to paint the volume of objects and the three-dimensional space of drawings. Thus, children aged 7–12 can represent proportional comparison features. Children need to learn and practice the expressing method of distance comparison.

## Discussion

Most children use the front view to create their drawings in terms of observation perspective. This result may be related to their observing and creative habits. The front view drawing is what observers perceive by the front of objects and figures (Rose and Jolley, 2020 and Lindstrom, 2021). This result shows that most children are more familiar with the frontal appearance features of objects and figures. The contents of children’s drawings are related to the things children observed (Wright, 2014; Latham and Ewing, 2018; Metin and Aral, 2020). Therefore, children’s observation habit is from the front of objects and figures to observe. Only a minority of children use other observational perspectives in their works. This result is because children have less creative experience in several types of observational perspectives. The participants’ first grade mathematics textbooks included related lessons to guide children in identifying different viewing perspectives (Research Group of National Mathematics Curriculum Standards for Compulsory Education, 2013). As a consequence, the participants in this test had the capacity to identify the types of observational perspectives. However, according to the analysis result the observation view show these children were less able to apply their knowledge from the mathematics curriculum to their drawing practice. The results of the observation view also showed that the small number of children’s drawings contained more than one type of view. The presence of mixed views may also result in children paying attention to the visual features of objects and figures (Goodnow, 2013; Yurumezoglu and Oztas Cin, 2019). However, children do not know methods to unify the different observation views of objects in their works. Therefore, in children’s painting creation education, teachers should guide children to observe the appearance features of objects from different observation views. Long-term observation training can help children distinguish the shape features of objects under different observation views and methods of drawing expression.

As for the baseline performance, there was an upward trend in the baseline performance ability of children from 7 to 10 years old. When children reach 11–12 years old stage, their baseline performance ability trend downward. This result may related to the habit of children’s drawing practice. Because children pay too much attention to the effects of painting creation, they may ignore the problems of painting skills in the painting process. The children who participated in the test had already learnt to judge the position and order of objects in their year 1 mathematics curriculum (Research Group of National Mathematics Curriculum Standards for Compulsory Education, 2013). However, they neglected to represent the skills they had learned in the mathematics curriculum in their work. When children accomplish the works, they may classify the creative experience as a successful creative experience. They may keep repeating the form of painting they think is correct. Children in this situation may repeatedly show weaknesses in their compositional and stylistic skills in the pieces. At the same time, children are unaware of the problems with drawing. Children’s drawing abilities such as composition, sketching, and color perception may not be improved by continuous painting practice. The results based on the baseline and comparisons in this study also demonstrate the problems of children’s drawing practice. The developmental stage of children’s drawing is from the 7–8 years old stage to the 9–10 years old stage. The visual effects of 9–10 years old children’s drawings show a decreasing trend due to the limitations of drawing ability and cognitive development level (Figure 1). Therefore, for children in the schematic and dawning realism stages, it is more important to help children develop the ability to identify problems in their drawings than continually complete their works.

As for the performance of comparison features, the fewest display comparison features in children’s drawings is distance comparison. Children’s drawing ability, observation ability, drawing habits, and spatial perception ability are the reasons that display minor distance comparison features in their works. The expression of distance comparison features in paintings is related to children’s cognition of positional (Mix, 2019). The performance of the distance comparison features in the drawings is connected to the children’s understanding of the concepts of occlusion relationship and proportional comparison relationship (Chu et al., 2018). The formation of the occlusion relationship is due to the existence of a front-to-back position relationship between two objects. Moreover, the object near the observer partially occludes the thing far from the observer. Comparison of proportions is related to the relationship between the distance and position of the objects. Objects that are far from the observer are smaller than objects that are close to the observer. The appearance of occlusions and distance comparisons are related to children’s drawing abilities. Children with well-drawing skills may focus on the location and proportions of objects. However, children with limited drawing ability may neglect to draw the proportional and positional features of the things. Thus, children’s drawing ability may become one factor that influences their ability to express the distance comparison feature. The appearance of objects depends on children’s observation of natural and living scenes. It is difficult for some children who have not received drawing training to create works using professional drawing techniques and perspective principles. This part of children will only develop drawings by observing objects in nature and life scenes. The children’s observation ability will determine the visual effect of their works. Children with a high level of observation ability can pay attention to changes in the proportions and shapes of objects from different viewing perspectives. However, children with weaker observation skills may ignore changes in the shape and characteristics of objects. Hence, the expression of distant comparison features is relevant to children’s observation ability. Some of the children who relied on imagination to create their works showed distant comparison features that probably related to their drawing habits. The drawings created by imagination lack the process of observing life scenes. The creative process of these children may consist of repeating their familiar drawing experiences. Children may not characterize objects according to drawing rules or real-life scenarios. These children also cannot draw distant comparison features. As a result, the painting habit will become a factor that restricts these children’s performance of distant comparison features. Furthermore, the proportional features of objects presented in children’s drawings are related to their quantity sense (Odic, 2018). This sensory ability develops as children visually estimate objects’ width, height, and volume. Children’s ability to assess objects’ quality is associated with mathematical learning (Atit et al., 2021). The participants also had volume sense and physical sense training in their second-grade mathematics curriculum (Research Group of National Mathematics Curriculum Standards for Compulsory Education, 2013). These children may have grasped methods of rough estimating the volume and physical features of objects. However, children’s ability to accurately assess the height, width, and depth features of objects requires further training.

## Conclusion

This study assesses children’s drawing based on viewing perspective, baseline, and comparison features. The observation perspective that children most apply is the front view. The result indicates that most children have the highest awareness of the spatial characteristics of the front of the object. Therefore, in the practice teaching of painting to children aged 7–12, art teachers should guide children to analyze the changes in visual characteristics of objects from different viewing angles (such as changes in length, width, height, and volume under different viewing angles) and position and position changes in features (such as occlusions between objects, changes in positional relationships such as neighbors and distances). Fine arts teachers also need to guide children to observe the changes in the shape and spatial from different viewing perspectives. In addition, teachers need to guide children not only limited in identifying different viewing perspectives of objects but also in analyzing changes in the contour features of objects from different viewing perspectives.

The appearance of baseline features shows that more than half of the children between the ages of 7 and 12 have the sense to show baseline features. This result also indicates that children have cognition of dividing the visual space area of the drawing and expressing the ground plane. Although more than half of the children were conscious of the baseline characteristics, children drew objects on the baseline. Therefore, children’s ability to accurately perform baseline performance still needs to be trained. The performance of baseline correlated with children’s ability to represent visuospatial regions. Therefore, the premise of baseline feature performance is to guide children to use the location features of different objects to paint the regional elements of the sky and the ground. For example, the location features of elements such as clouds, sun, moon, and birds represent the regional features of the sky. Use the location elements such as plants, houses, and people to express the regional characteristics of the ground. At the same time, fine arts teachers can guide children to identify the position of the apparent horizon and horizon in real-life scenes. The horizon is the mark that divides the sky and ground areas in the drawing. The position of the apparent horizon determines the viewing angle of a person. If children display the position features of apparent horizon and horizon, they are likely to accurately characterize areas of the ground and sky in their drawings. Therefore, the position training of observing the horizon and eye level is an effective method to improve the characteristics of different visual areas of children’s performance works.

Children’s ability to draw distant comparison features remains to be improved. The proportional features between objects are related to the length and width features of the objects. It indicates that children have well able to express the length and width features of objects. The comparison of distant features in the drawing relates to expressing the spatial depth of the volumetric features. The children aged 7–12 can display the visual effects of objects in a flat. However, the ability of these children to express the three-dimensional visual effect of works still needs further training. The key to improving three-dimensional visual space ability is to establish children’s cognition of the volume of objects and the cognition of position. When guiding children’s drawing practice, fine arts teachers need to provide children with the methods of visualizing objects’ contour and volume features. For instance, fine arts teachers can assist children in achieving their ability to visualize contour features by observing the length and width features of objects. Children’s ability to represent volumetric features can be facilitated by directing their attention to the height and depth of the object in space. Meanwhile, teachers need to instruct children to observe the changes in the contours and volume features of the objects with different position relationships. Therefore, the development of the ability to express comparison features achieves by training children’s ability to draw the volumetric and positional features of objects in their drawings.

The above results indicate that children’s awareness of applying interdisciplinary knowledge in practical drawing creation activities is weak. Children have only grasped the estimation methods in the mathematics curriculum related to estimating the features of length, width, and volume of objects. However, children’s ability to represent the geometric features from visual assessment in their drawings still needs to be improved. Teachers need to guide children to observe the features of objects in real-life situations. Some teachers may only use images to teach children to estimate the geometric characteristics of objects. As a result, children will be unable to accurately understand the width, height, and volume of objects in real-life scenes. Therefore instructing children to observe the geometric and spatial features of objects in real-life situations needs to be actually integrated into the fine arts curriculum.

## Data availability statement

The raw data supporting the conclusions of this article will be made available by the authors, without undue reservation.

## Ethics statement

The studies involving human participants were reviewed and approved by Beijing Normal University Ethics Committee. The patients/participants provided their written informed consent to participate in this study.

Written informed consent was obtained from the individual(s) for the publication of any potentially identifiable images or data included in this article.

## Author contributions

LY contributed to the conceptualization, methodology, formal analysis, investigation, data curation, and draft writing. YL is responsible for reviewing and editing the article. All authors contributed to the article and approved the submitted version.

## Conflict of interest

The authors declare that the research was conducted in the absence of any commercial or financial relationships that could be construed as a potential conflict of interest.

## Publisher’s note

All claims expressed in this article are solely those of the authors and do not necessarily represent those of their affiliated organizations, or those of the publisher, the editors and the reviewers. Any product that may be evaluated in this article, or claim that may be made by its manufacturer, is not guaranteed or endorsed by the publisher.
